# Supplementary material for: Insight and inference for DVARS
Source: Neuroimage. 2018 May 15;172:291–312. doi: 10.1016/j.neuroimage.2017.12.098 (PMC5915574; doi:10.1016/j.neuroimage.2017.12.098)
Supplement: mmc1 [file mmc1.pdf]

Supplementary Material for: Insight and Inference for DVARS

Soroosh Afyouni[a,b,c], Thomas E. Nichols[a,d,]

[a] *Oxford Big Data Institute, Li Ka Shing Centre for Health Information and Discovery, Nuffield Department of Population Health, University of Oxford, Oxford, OX3 7LF, UK*
[b] *Institute for Advanced Studies, University of Warwick, Coventry, CV4 7AL, UK*
[c] *Institute for Digital Healthcare, WMG, University of Warwick, Coventry, CV4 7AL, UK*
[d] *Wellcome Centre for Integrative Neuroimaging, FMRIB, Nuffield Department of Clinical Neurosciences, University of Oxford, Oxford, OX3 7LF, UK*

1. DSE Table

Table S1: Make up of the DSE table giving a mean squared (MS) SS decompositions of resting-state fMRI data. The first row shows how the total MS can be split into 3 terms, in the second through 4th columns, $A = D + S + E$. The first column likewise shows how total MS can be decomposed in to that explained by a spatially global time series (second row) and a non-global or residual-global component (third row), $A = A_G + A_N$. Likewise, each row and column sums accordingly: $A_G = D_G + S_G + E_G$, $D = D_G + D_N$, etc. Terms are shown here as MS for brevity, but are best reported in root mean squared (RMS) units. See Table 1 for definitions of the time series variables.

	A-var	D-var	S-var	E-var
Whole	$A = \frac{1}{T}\sum_{t=1}^{T} A_t$	$D = \frac{1}{T}\sum_{t=1}^{T-1} D_t$	$S = \frac{1}{T}\sum_{t=1}^{T-1} S_t$	$E = \frac{1}{T}\sum_{t=1,T} E_t$
Global	$A_G = \frac{1}{T}\sum_{t=1}^{T} A_{Gt}$	$D_G = \frac{1}{T}\sum_{t=1}^{T-1} D_{Gt}$	$S_G = \frac{1}{T}\sum_{t=1}^{T-1} S_{Gt}$	$E_G = \frac{1}{T}\sum_{t=1,T} E_{Gt}$
Non-Global	$A_N = \frac{1}{T}\sum_{t=1}^{T} A_{Nt}$	$D_N = \frac{1}{T}\sum_{t=1}^{T-1} D_{Nt}$	$S_N = \frac{1}{T}\sum_{t=1}^{T-1} S_{Nt}$	$E_N = \frac{1}{T}\sum_{t=1,T} E_{Nt}$

2. Datasets

Here we provide additional information on the HCP and PCP subjects considered, including subject IDs and basic demographics. Detailed evaluation with DSE plots and ANOVA tables are presented for two subjects per cohort.

Email addresses: `soroosh.afyouni@bdi.ox.ac.uk` (Soroosh Afyouni), `thomas.nichols@bdi.ox.ac.uk` (Thomas E. Nichols)

2.1. HCP Subjects

The intensity of both minimally and fully pre-processed datasets downloaded from the HCP repository have been normalised to 10,000 by default (see Glasser et al. (2013) and the HCP documentation on `http://www.humanconnectome.org/documentation/`; visited on January 24th 2017). Therefore, we scale the minimally and fully pre-processed datasets by 1/100, according to Eqn. 1, to reach the intensity 100.

We use the *100 Unrelated Subjects* standard package on the HCP data-base, access on July 2017. Only the first sessions with Left-Right(LR) phase-encoding was downloaded. Further to these 100 subjects, we also use two subjects throughout the paper to demonstrate how they behave to the method developed in this work. They are listed as below:

Table S2: List of subjects from the **HCP** cohort. Subject IDs in bold are among the ones featured in the main text.

Subject ID	Gender	Age Range (Year)	Mean FD (mm)	Phase decoding direction	Session
**115320**	F	31-35	0.204	LR	1
**118730**	M	22-25	0.166	LR	1

2.2. ABIDE Subjects

Similarly, for the ABIDE-NYU cohort, we follow instruction given on the PCP website (`http://preprocessed-connectomes-project.org/abide/Pipelines.html`; visited on October 17th 2016). No intensity normalisation has been reported for minimally pre-processed datasets while intensity of the fully pre-processed datasets normalised by 1,000. Therefore, we normalise the intensity of minimally pre-processed datasets according to Eqn. 1 (m^R as intra-mask median of the mean image) and scale the pre-processed datasets by 1/10 to reach our desired typical intensity value of 100. The ABIDE fully preprocessed data is provided as mean-zero 4D data and a separate mean image. We note that, for the ABIDE subjects listed in Table S3, none appeared had mean intensities around 1,000. Specifically, for the two subjects that we investigate in the next section (51050 and 51055), the median of the mean brain is found to be 66.80 and 68.076, respectively; however, scaling by (e.g.) $1/66.80\times100$ led to less realistic values, and hence we have chosen to use the documented mean value and scale the ABIDE fully pre-processed data by 1/10.

Table S3: List of subjects from the **ABIDE - NYU** cohort.

Subject ID	Gender	Age (Year)	Mean FD (mm)
51036	F	8.04	0.103
51038	F	8.26	0.060
51039	F	8.5	0.089
51040	F	8.52	0.037
51041	F	8.9	0.070
51042	F	8.91	0.117
51044	F	10.86	0.076
51045	F	11.56	0.151
51046	F	11.56	0.076
51047	F	12.1	0.055
51048	F	12.44	0.078
51049	F	13.22	0.071
51050	F	13.25	0.056
51051	F	14.06	0.114
51052	F	14.18	0.067
51053	F	14.38	0.072
51054	F	14.42	0.146
51055	F	15.95	0.077
51056	F	17.31	0.032
51057	F	21.58	0.052
51058	F	22.11	0.091
51059	F	22.76	0.064
51060	F	22.74	0.168
51061	F	27.03	0.040
51062	F	27.76	0.096

2.3. DVARS Inference

Table S4: List of all statistically significant spikes on the fats component, D-var, of minimally pre-processed HCP 118730. Spikes which represent the highest (index 7) and lowest (index 726) are marked in bold. Similar analysis presented in Table 5 for HCP subject 115320.

Scan	Index	DVARS	$\sqrt{\text{D-var}}$	%D-var	Δ%D-var	RDVARS	Z(D-var)	FD
6 & 7	6	6.146	3.073	39.926	10.969	1.171	9.673	0.200
**7 & 8**	**7**	**8.148**	**4.074**	**70.155**	**41.198**	**1.552**	**36.330**	**0.384**
8 & 9	8	7.811	3.905	64.477	35.520	1.488	31.323	0.345
9 & 10	9	5.687	2.843	34.179	5.222	1.083	4.605	0.243
36 & 37	36	6.057	3.028	38.766	9.809	1.154	8.650	0.301
37 & 38	37	5.938	2.969	37.269	8.312	1.131	7.330	0.163
268 & 269	268	6.420	3.210	43.557	14.600	1.223	12.875	0.366
269 & 270	269	6.249	3.124	41.275	12.318	1.191	10.862	0.111
426 & 427	426	6.241	3.120	41.158	12.201	1.189	10.760	0.412
427 & 428	427	6.015	3.007	38.238	9.281	1.146	8.185	0.242
573 & 574	573	5.690	2.845	34.211	5.254	1.084	4.634	0.046
575 & 576	575	5.742	2.871	34.838	5.882	1.094	5.186	0.257
576 & 577	576	5.701	2.850	34.349	5.392	1.086	4.755	0.378
615 & 616	615	5.695	2.847	34.279	5.322	1.085	4.693	0.278
652 & 653	652	5.967	2.983	37.633	8.676	1.137	7.651	0.465
653 & 654	653	6.179	3.089	40.353	11.396	1.177	10.049	0.219
**726 & 727**	**726**	**5.664**	**2.832**	**33.905**	**4.948**	**1.079**	**4.364**	**0.433**
783 & 784	783	6.431	3.215	43.709	14.752	1.225	13.009	0.253
784 & 785	784	6.402	3.201	43.319	14.362	1.220	12.665	0.156

3. Simulations

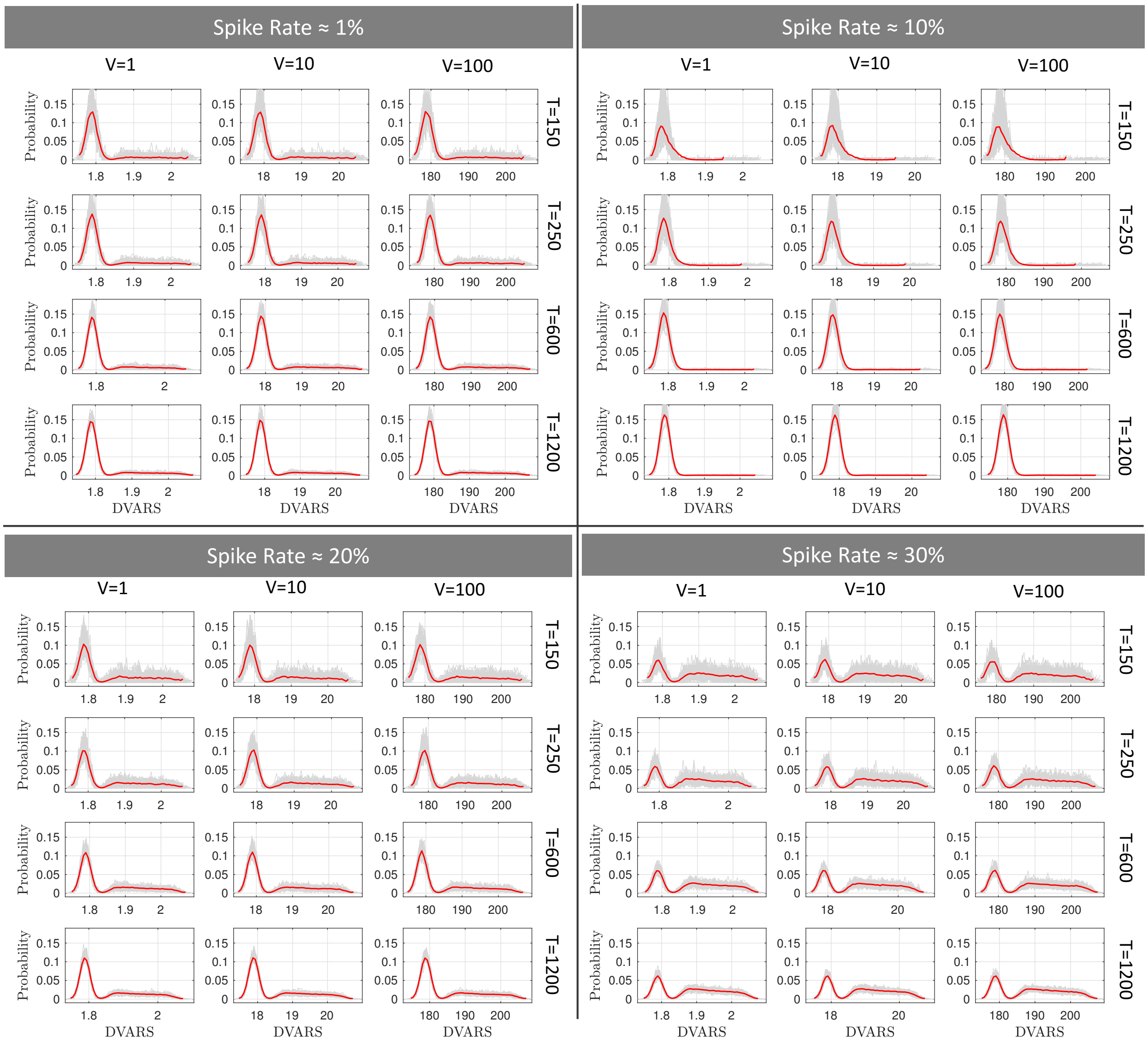

Figure S1: DVARS histograms with different time series lengths (rows, within panel) and different variance heterogeneity (left to right, within panel). The grey shades show the realization of DVARS simulations and solid read line indicate the mean of the these realisations. Each panel illustrates DVARS simulations for shown spike rate. Spike rate is percentage time series length as spikes.

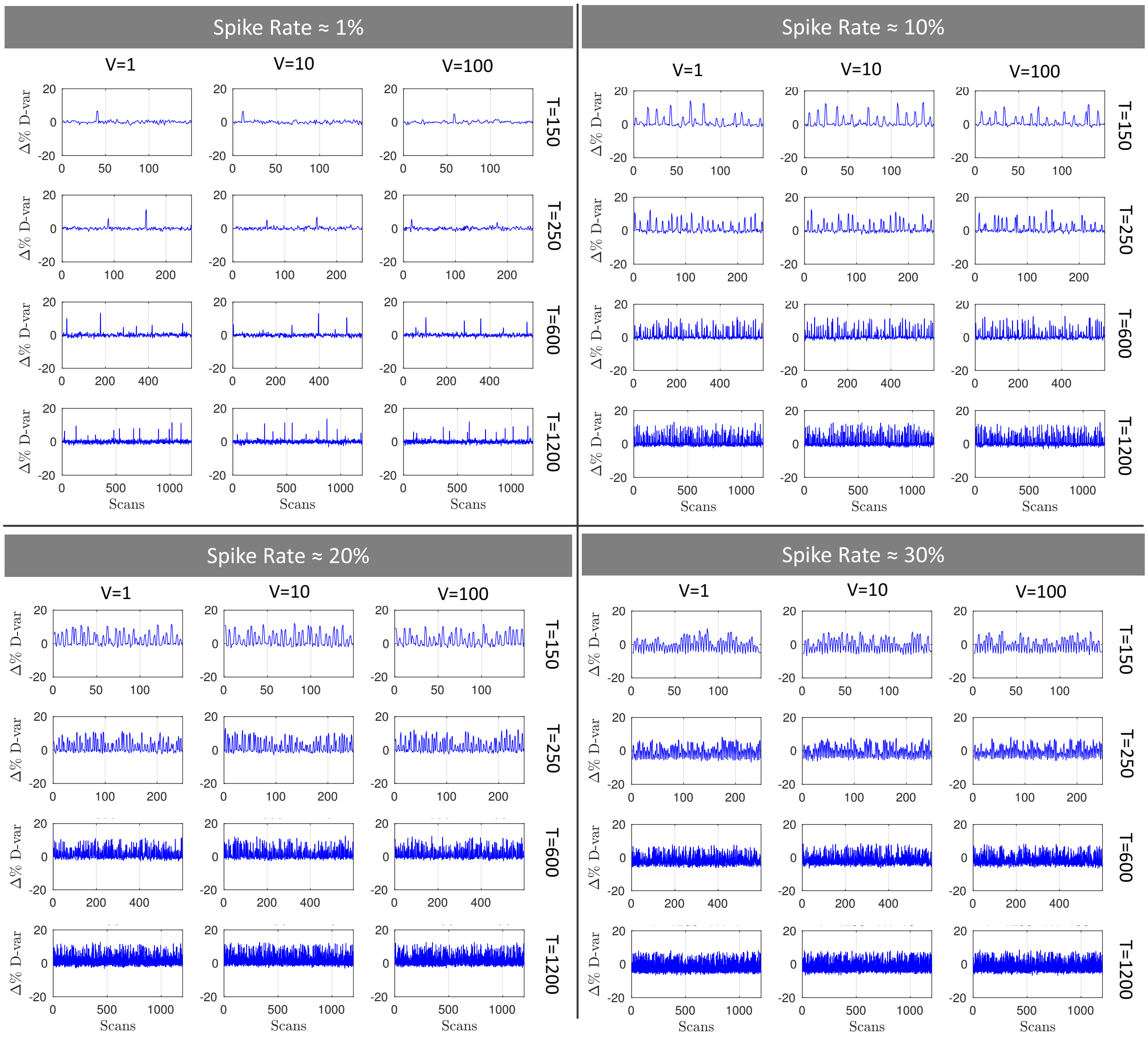

Figure S2: Examples of DVARS simulations with different time series lengths (rows, within panel) and different variance heterogeneity (left to right, within panel). See Section 3.1 for more details about simulation settings. In this example, the autocorrelation coefficient is $\rho = 0.4$

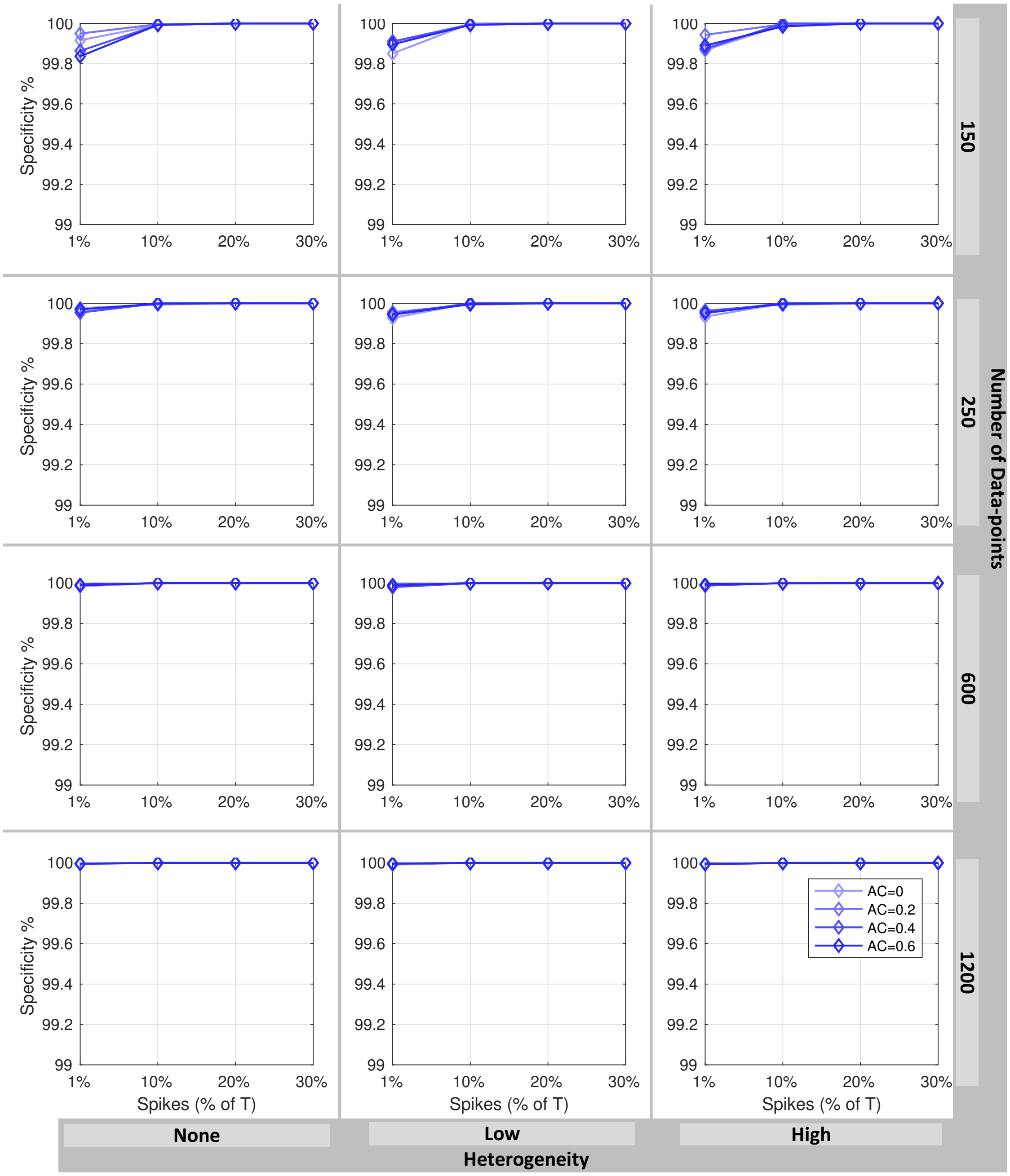

Figure S3: Specificity of DVARS tests with different time series lengths (rows) and different variance heterogeneity (columns). The specificity of each condition was shown across different number of spikes (as percentage of sample size) and different level of autocorrelations (solid line with shades of blue.)

4. Standardised DVARS

4.1. Results for HCP

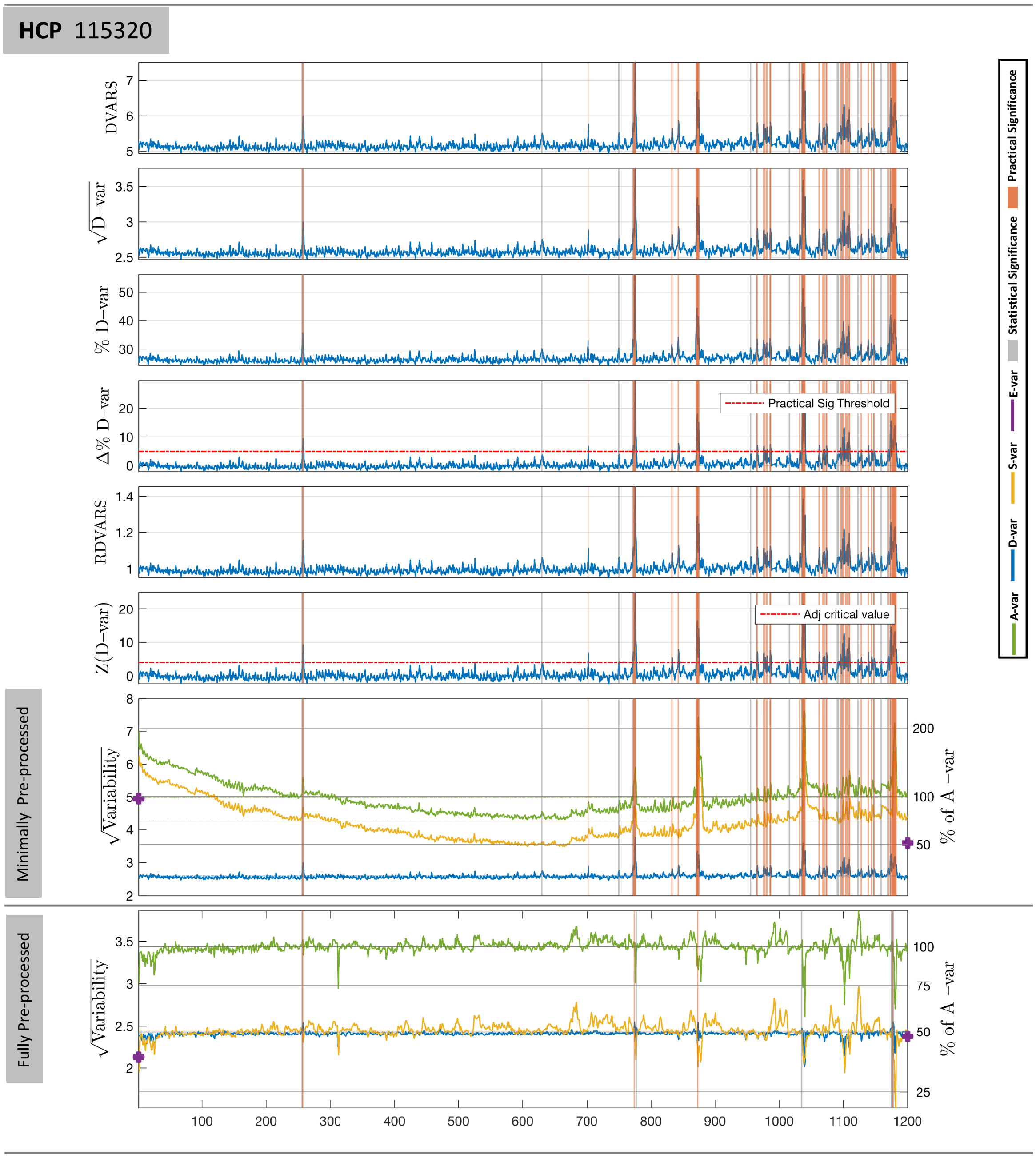

Figure S4: Same as Figure 4, except for HCP subject 51055. Here, note how $\Delta\%D$-var ranges up to 30% in the minimally pre-processed data, while in the fully Pre-processed data (very bottom plot), D-var never exceeds about 55% (i.e. 5% $\Delta\%D$-var). Hence while the post-clean-up data still has some significant DVARS scans, the severity of each is much reduced.

4.2. Results for ABIDE-NYU

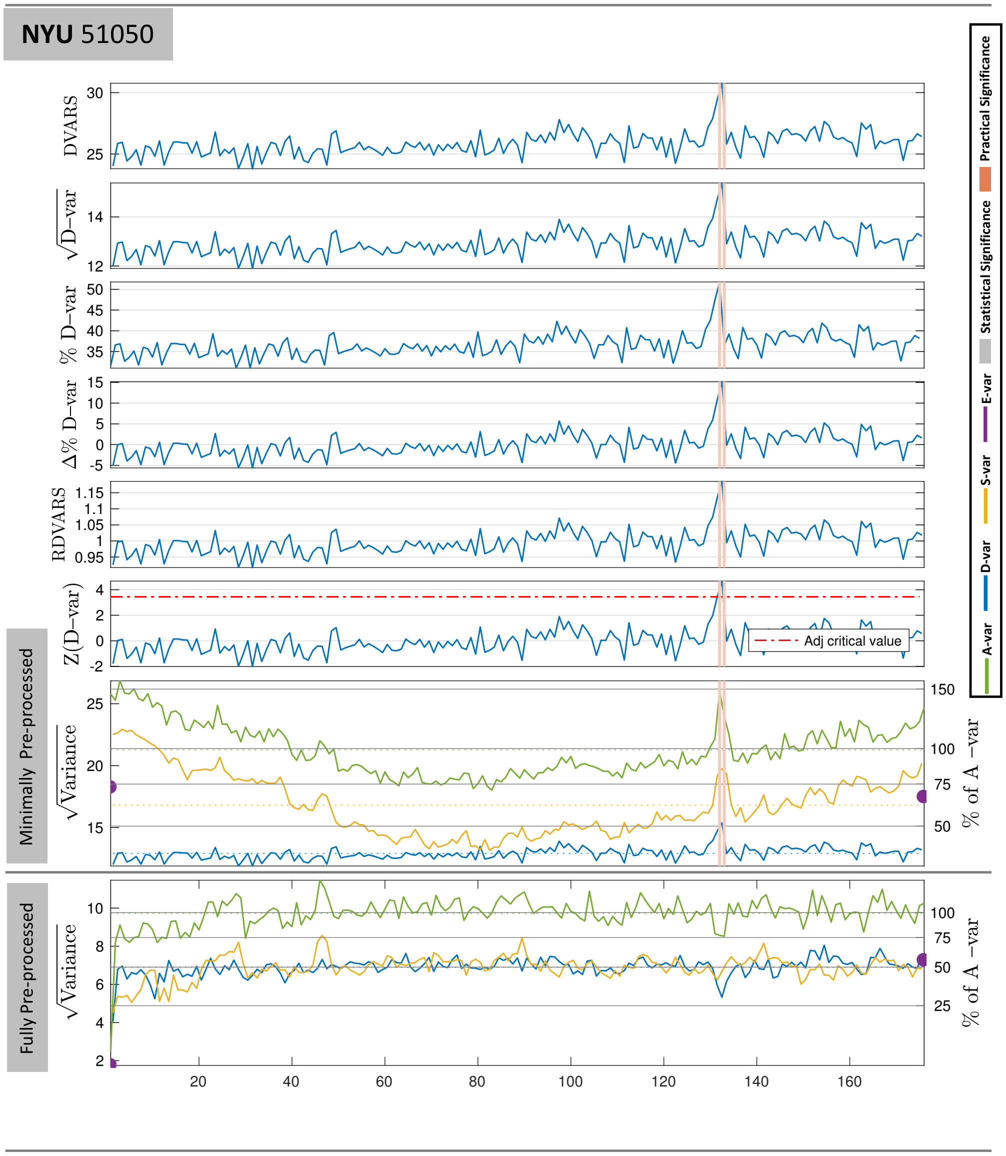

Figure S5: Comparison of different variants of DVARS-related measures on ABIDE-NYU 51050. The same format was used as Figure 4. For minimally pre-processed dataset, several spikes were identified as statistically significant between scan 130 to 140. These spikes were eliminated by regressing out the motion parameters (in the fully pre-processed dataset) as shown in the very bottom sub-figure. The bottom sub-figure also illustrates how the contribution of the slow and fast variability components has converged (with more reduction in the slow variability) to 50% in the fully pre-processed dataset.

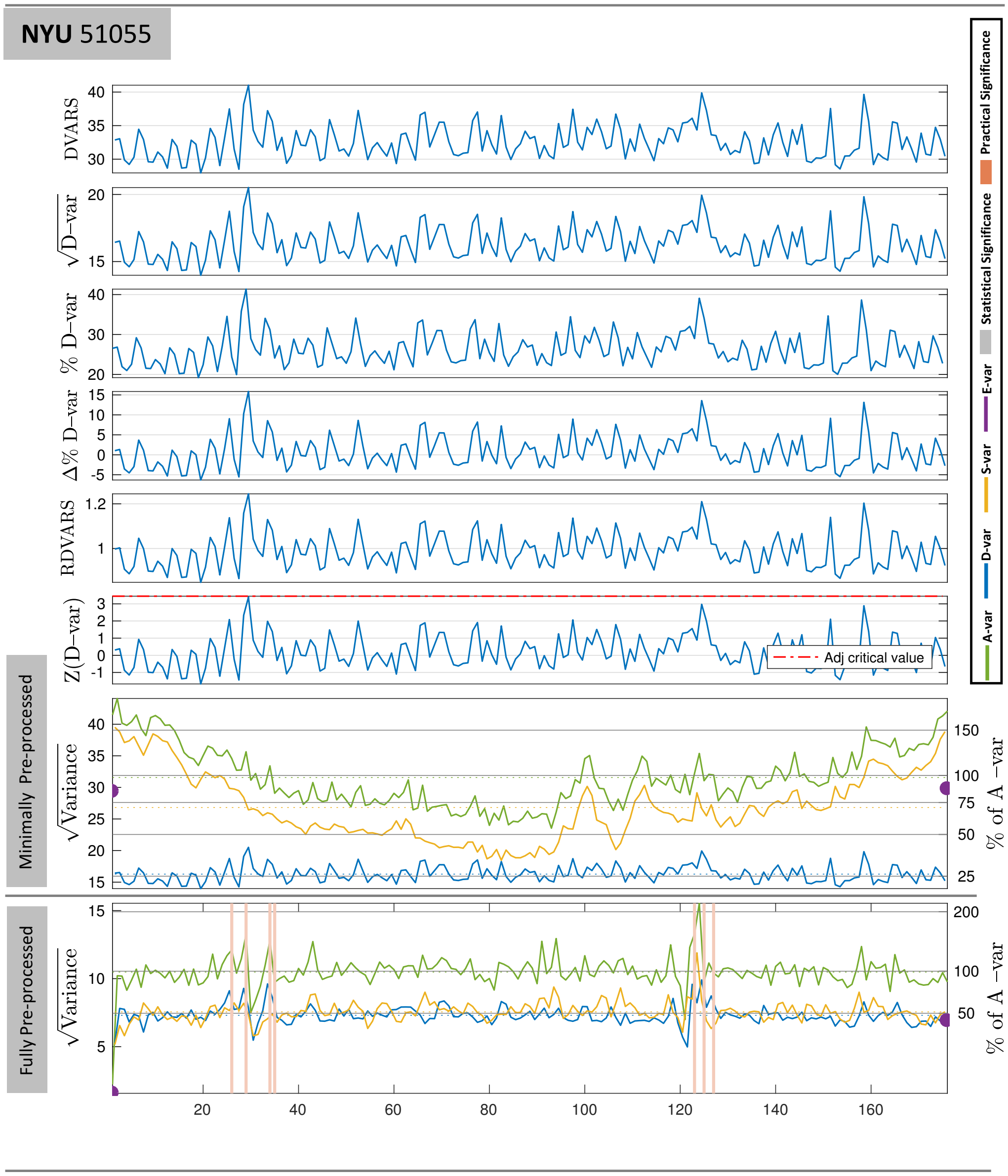

Figure S6: Comparison of different variants of DVARS-related measures on ABIDE-NYU 51055. The same format was used as Figure 4. For minimally pre-processed data set the average variability is found to be ≈ 32, while in fully pre-processed stage, average variability falls to ≈ 10. With the dramatic reduction of noise there is improved power to detect spikes: While no significant spikes were found in minimally preprocessed data, fully pre-processed data (bottom sub-figure) has several significant spikes, some with D-var approaching 90% of A-var (corresponding to a Δ%D-var of %40).

5. Standardised DVARS Distribution

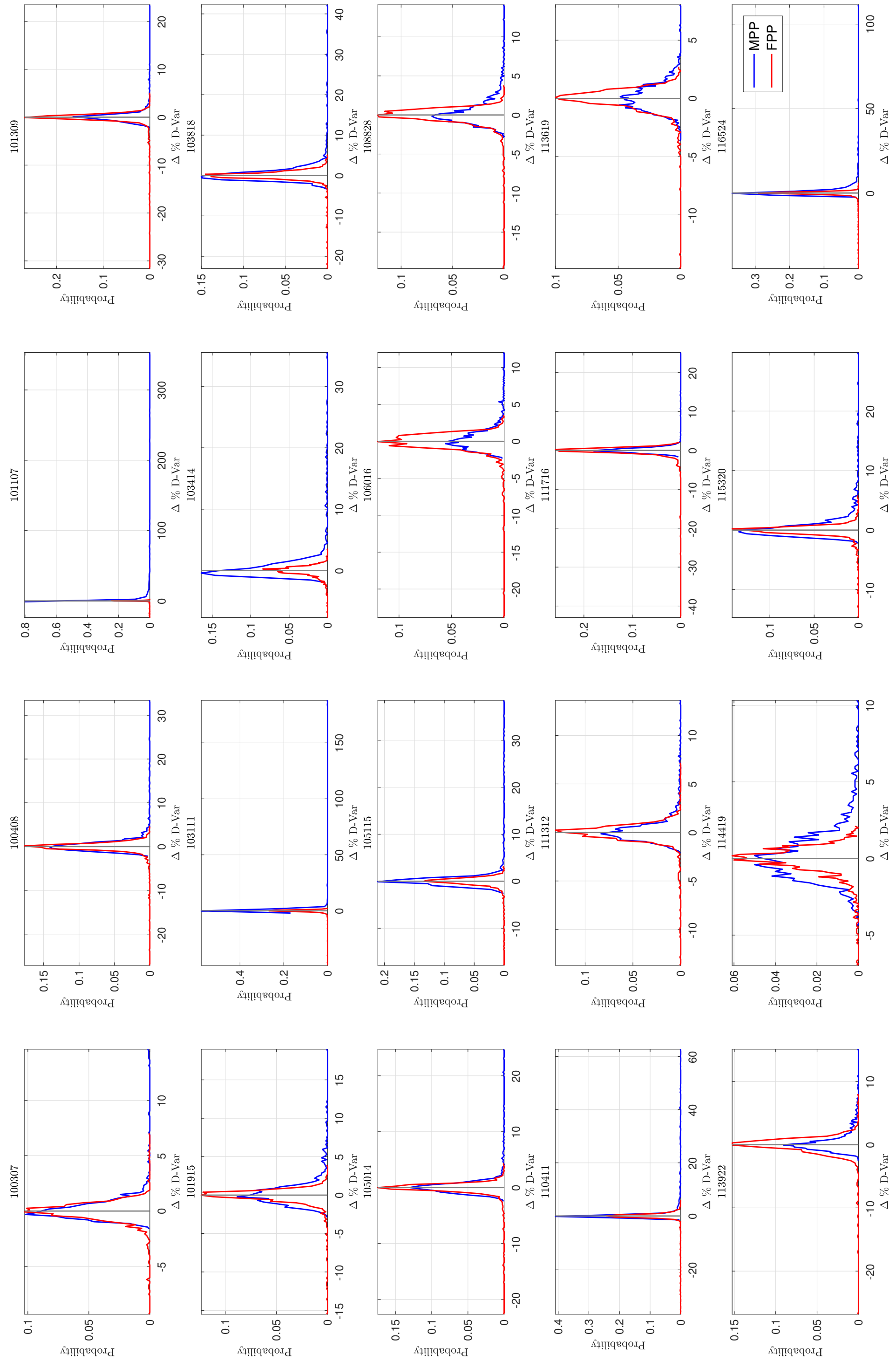

Figure S7: Empirical density estimate of DVARS for first 20 subjects in the HCP cohort.

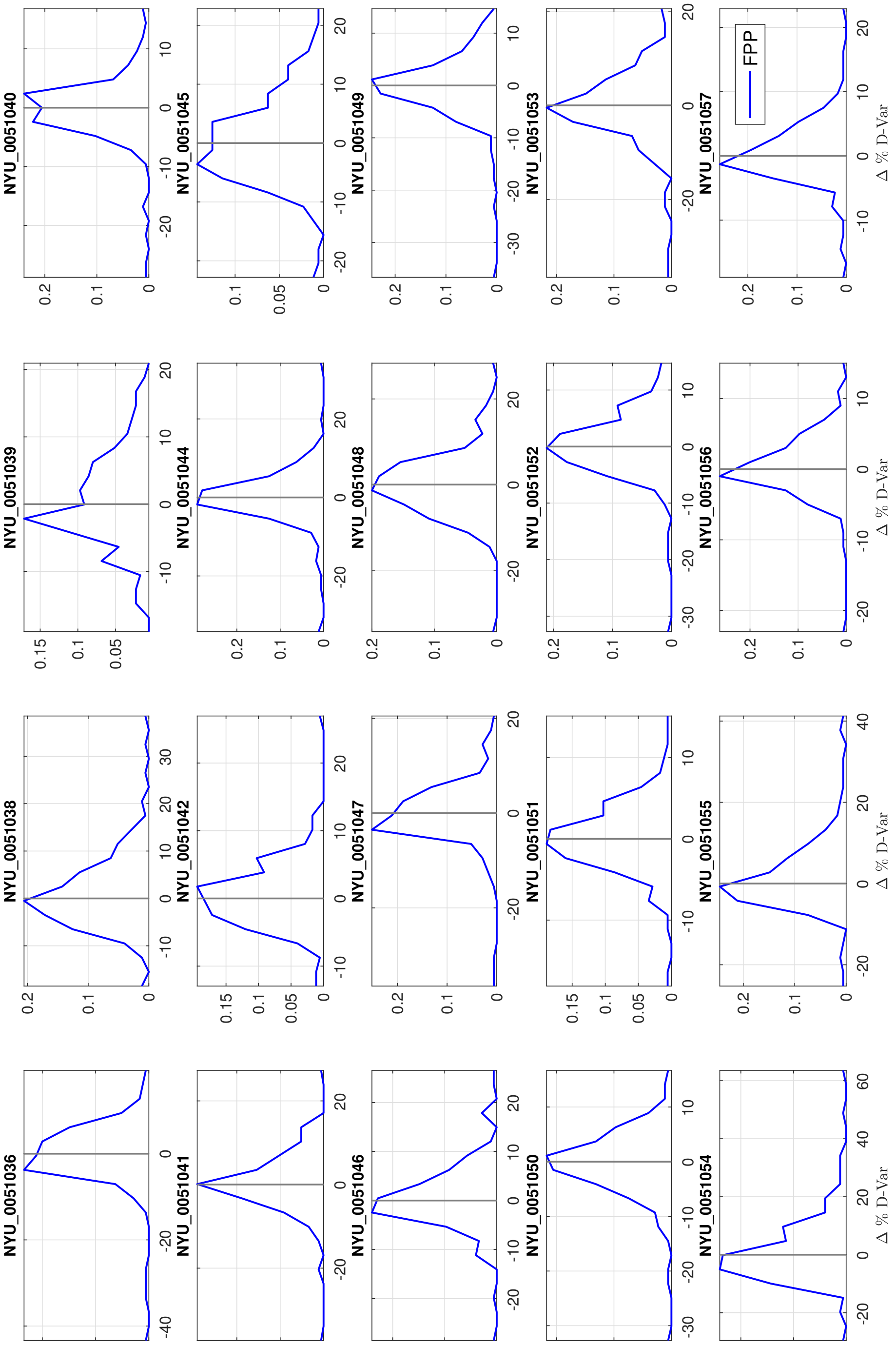

Figure S8: Empirical density estimate of DVARS for first 20 healthy subjects in the ABIDE-NYU cohort.

6. Effect of DVARS inference on Functional Connectivity

6.1. *Power Atlas*

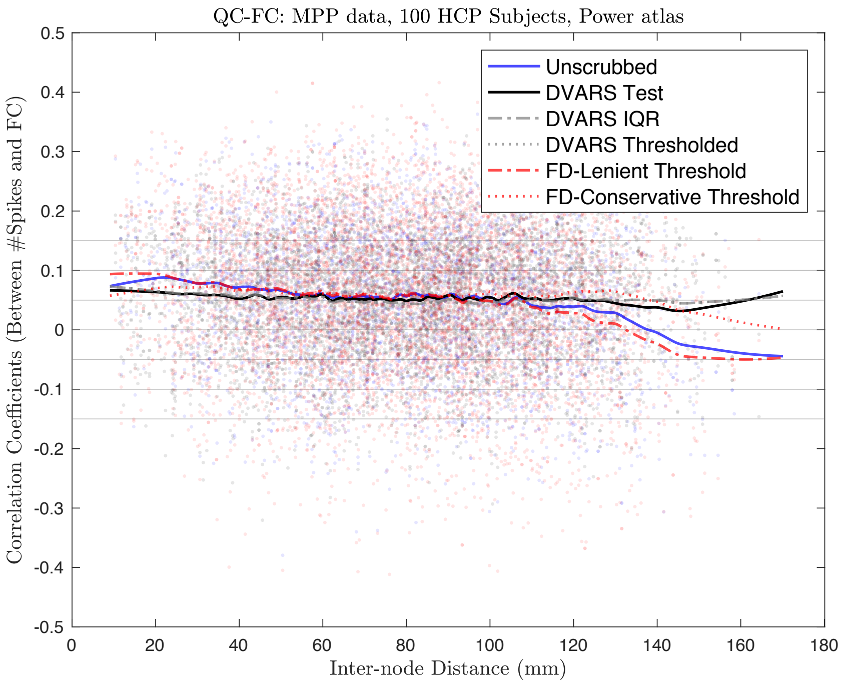

Figure S9: The QC-FC plots for five different FD/DVARS thresholding methods of 100 HCP subjects parcellated by Power Atlas Power et al. (2011). The transparent points indicate the association between portion of the volumes identified as corrupted (Burgess et al., 2016) by each method and inter-nodal distance (in mm). The horizontal lines indicate the LOESS smoothed fit to points of each method. The span window used for the LOESS algorithm is 1% of the whole data. The DVARS inference testing proposed in this work is marked as a solid black line. Note that a similar plot but for Gordon's atlas (Gordon et al., 2014) is presented in Figure 6 of the main text.

6.2. *Effect of DVARS testing vs. Random Scrubbing*

In this section, we compliment our FC analysis, in section XX, with FC analysis of random scrubbing described in Power et al. (2012, 2014); Burgess et al. (2016); Ciric et al. (2016).

6.2.1. *Methods*

We randomly remove the same number of spikes as detected via DVARS test. This procedure is repeated 1,000 times, forming a null distribution. A Z-score is computed for each edge, of FC computed after DVARS hypothesis test scrubbing compared to the null distribution, and statistically significant are assessed at α-level=5%, corrected for multiple comparisons via Bonferroni. the results are presented Gordon and Power parcellation schemes.

6.2.2. *Results*

Figure 6.2.2 shows that removal of DVARS-significant spikes results in significant changes in FC. Panel A and C of the figure shows the association between the changes in correlation coefficients, $\Delta r = FC(unscrubbed) - FC(scrubbed)$, and the distance between the corresponding pairs of ROIs (in blue). For both subjects removing significant spikes considerably changes FC, more so than random spike removal. To compare DVARS scrubbing to chance an edge-specific , at each edge, a time points equal towere randomly scrubbing a number of time points equal to the number of significant DVARS tests, we identify edges where DVARS-scrubbing is significantly different from random scrubbing.

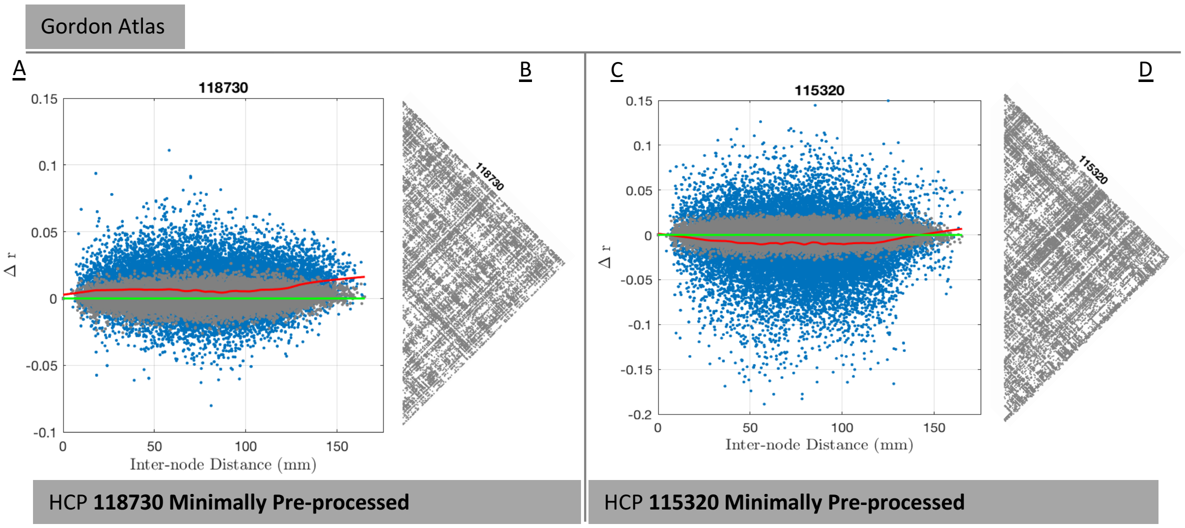

Figure S10: Changes in correlation coefficient of individual subjects due to scrubbing with the DVARS test. Panel A and Panel C, shows the changes in correlation coefficients of all 55,278 possible edges of Subject 18730 and 115320, respectively, with DVARS test scrubbing. The red solid lines indicate the smoothed fit for the empirical Δr and green solid lines indicate the the smoothed line for the null Δr. Panel B and Panel D, illustrate the edges which have been significantly affected due to censoring the identified volumes. In both subjects, a large number of edges have their FC changed significantly with DVARS test scrubbing.

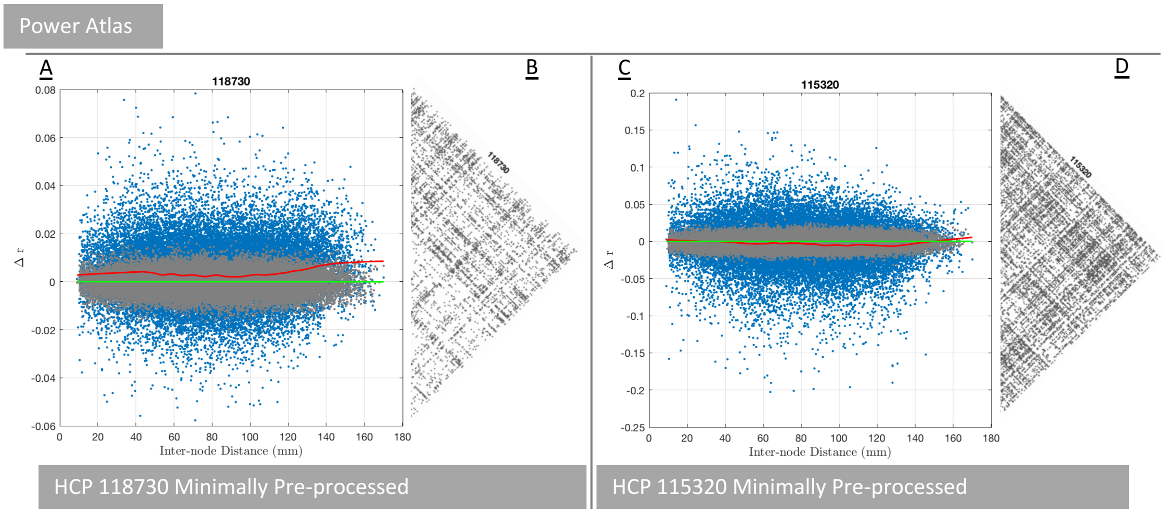

Figure S11: Panel A and Panel C illustrate the association between changes in correlation coefficients (Δr) of subject 118730 and 115320, respectively. The red solid lines indicate the LOESS smoothed lines for the empirical Δr and green solid lines indicate the the LOESS smoothed line for the null Δr. Panel B and D illustrate the edges which significantly changed due to DVARS inference testing.

7. Temporal Diagnostics: Minimally Pre-processed vs. Fully Pre-processed

7.1. Results for HCP

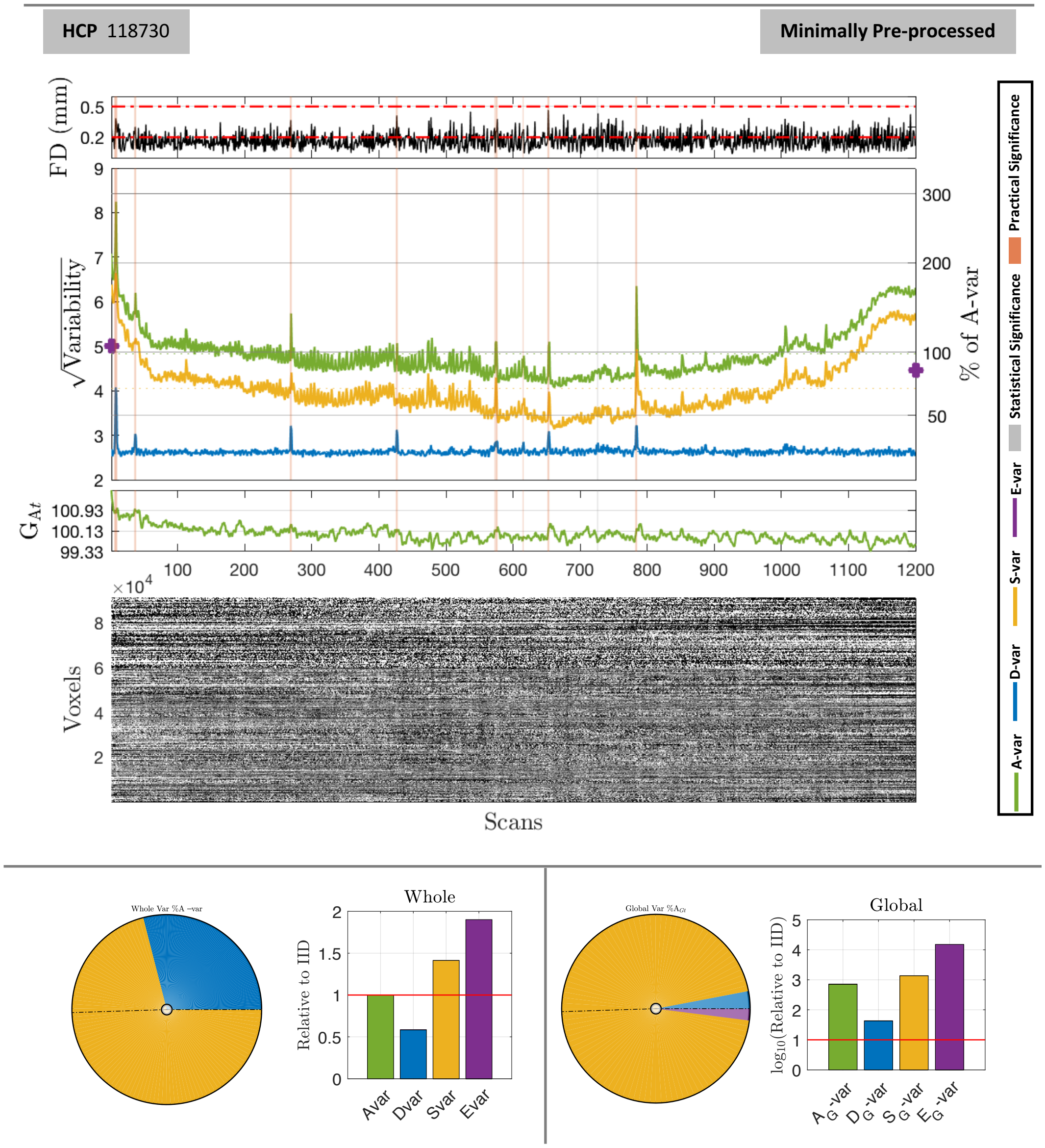

Figure S12: The DSE and DVARS inference for HCP subject 118730 minimally pre-processed. Layout as in Figure 5. As with the other HCP subject, the slow variability dominates in minimally pre-processed data. In contrast to subject 115320, note here the pie chart for the global reflects a larger D-var contribution, likely due to the high-frequency variation seen in the global (and total) time series.

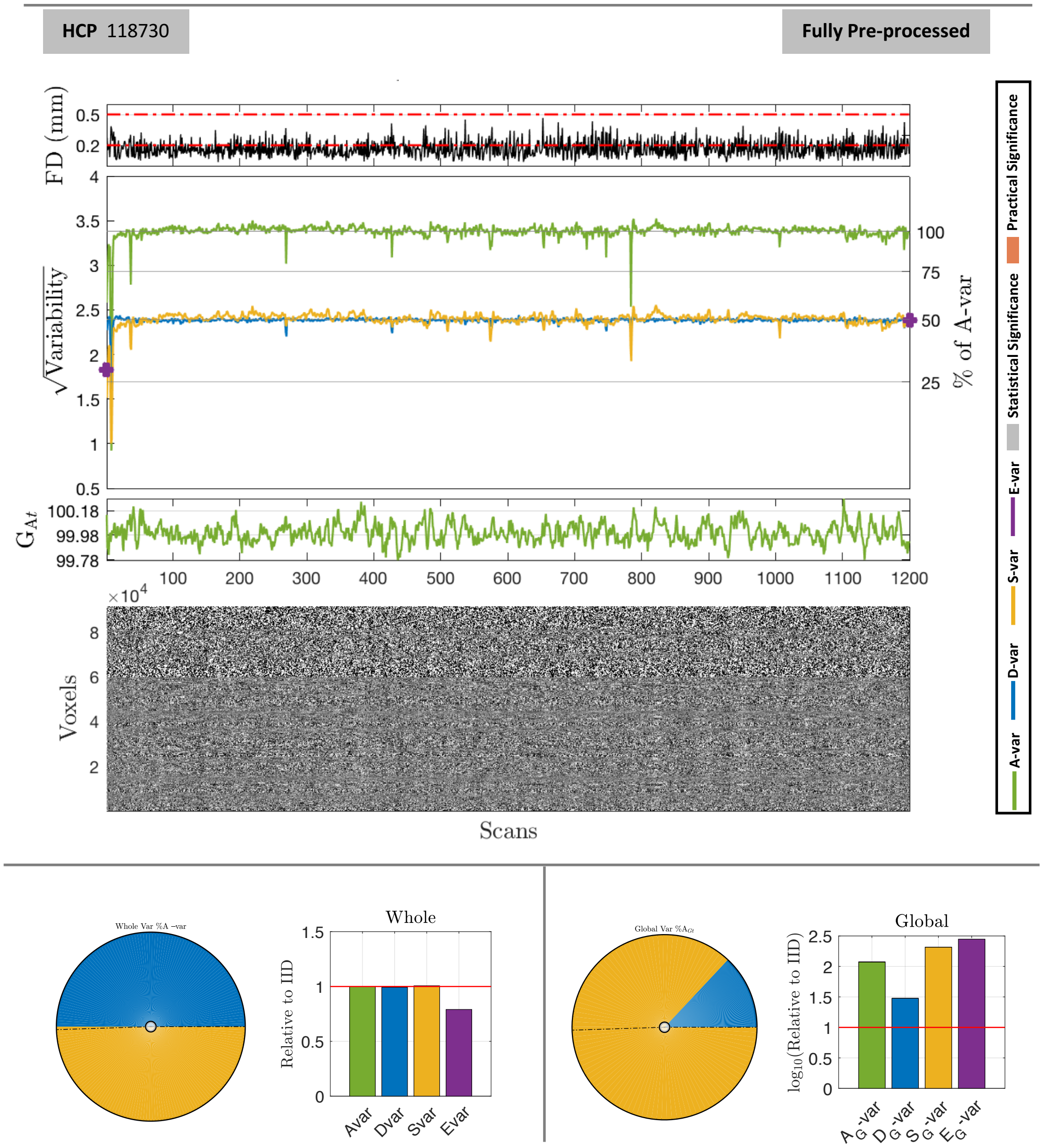

Figure S13: The DSE and DVARS inference for HCP subject 118730 fully pre-processed. Layout as in Figure 5. Previously fast D-var was around 2.5 RMS, and now has dropped to just under 2.5 and is matched by slow, S-var, variabilities. The global still reflects a relatively large D-var contribution, again due to the high-frequency variation seen the global time series.

Table S5: DSE ANOVA Tables for HCP 118730. Minimally preprocessed data (top), fully preprocessed (bottom) with similar format as Table 6. The RMS for slow variability in minimally pre-processed dataset, 4.09, is reduced to 2.4, more than 40% reduction. However, the same metrics for the fast variability is only increased for <9%. Also note the changes in the global share of the slow variability (S_G), which remarkably falls by 73% going from 0.265 to 0.071 while the global share of the fast variability (D_G) falls by ≈ 41% going from 0.265 to 0.071

Minimally Preprocessed Data

Source	RMS	% of A-var	Relative to IID
A - All	4.864	100.000	1.000
D - Fast	2.629	29.218	0.584
S - Slow	4.087	70.623	1.413
E - Edge	0.193	0.158	1.901
A_G - All Global	0.271	0.311	719.437
D_G - Fast Global	0.046	0.009	43.093
S_G - Slow Global	0.265	0.297	1,371.78
E_G - Edge Global	0.035	0.005	15,109.66

Fully Preprocessed Data

Source	RMS	% of A-var	Relative to IID
A - All	3.379	100.000	1.000
D - Fast	2.381	49.649	0.993
S - Slow	2.396	50.284	1.006
E - Edge	0.086	0.065	0.790
A_G - All Global	0.076	0.051	118.817
D_G - Fast Global	0.027	0.006	29.960
S_G - Slow Global	0.071	0.044	207.4062
E_G - Edge Global	0.003	< 0.001	280.0416

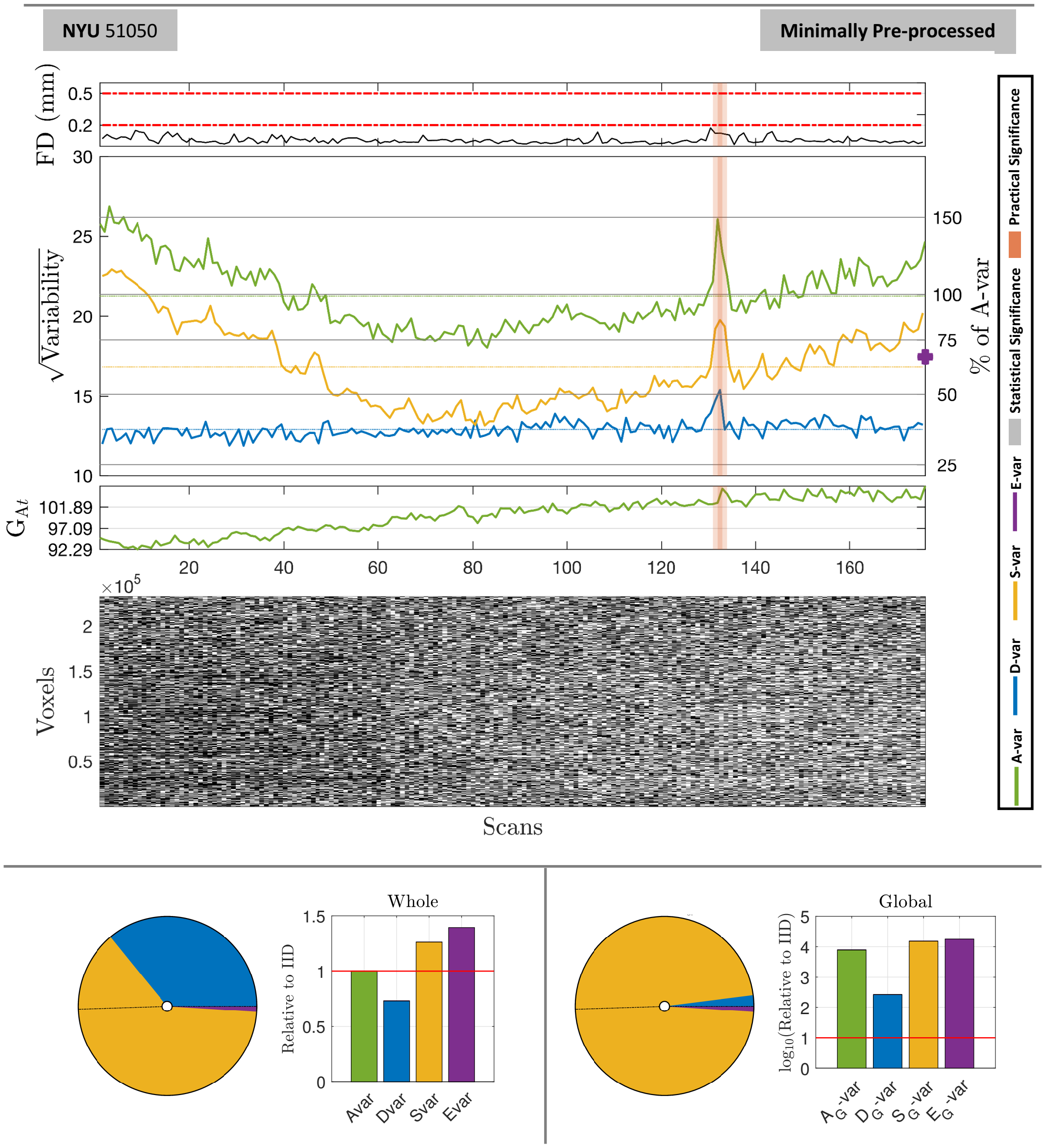

Figure S14: The DSE and DVARS inference for ABIDE-NYU 51050 minimally pre-processed. Layout as in Figure 5. As with the other ABIDE-NYU and HCP subjects, the slow variability dominates in minimally pre-processed data. Surprisingly, the statistically significant scan, marked with grey strip, has never exceeded any of the FD thresholds, despite $\Delta\%D$-var value in Figure S5 suggests more than 15% exceed.

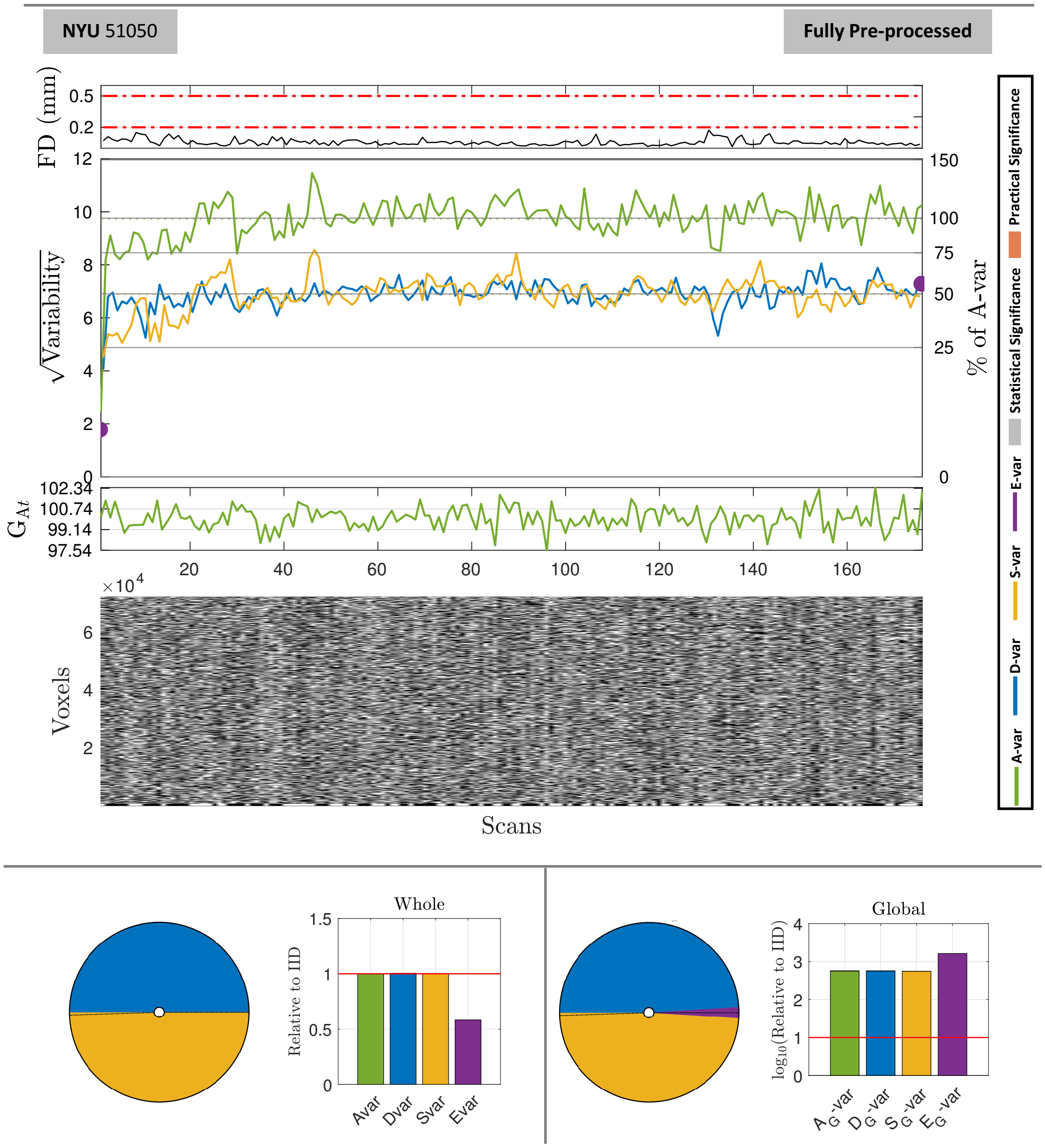

Figure S15: The DSE and DVARS inference for ABIDE-NYU 51050 fully pre-processed level. Layout as in Figure 5. In contrast to minimally preprocessed case for the same subject, here the pie chart for the global reflects a relatively larger D-var contribution, likely due to the higher level of high-frequency variation seen the global (and total) time series (compare the fluctuations in the third sub-figure of Figure S5 and Figure S6).

Table S6: DSE ANOVA Tables for ABIDE-NYU 51050. Minimally preprocessed data (top), fully preprocessed (bottom) are readily compared: In minimally pre-processed dataset, the S-var contribution is larger than the D-var contribution (36.36% and 62.85%, respectively). In fully pre-processed dataset, these contributions are almost equal (49.9% and 49.8%, respectively). It is worth noting, as shown in other subjects across both cohorts, the S-var experiences a larger reduction in its RMS than D-var. Interestingly, compared to the HCP cohort, RMS of global variabilities are larger, mainly due to high variability in the global part of the slow variability. Although this variability is reduced remarkably after full pre-processing.

Minimally Preprocessed Data

Source	RMS	% of A-var	Relative to IID
A - All	21.377	100.000	1.000
D - Fast	12.890	36.362	0.731
S - Slow	16.946	62.845	1.264
E - Edge	1.902	0.791	1.393
A_G - All Global	3.927	3.375	7873.420
D_G - Fast Global	0.507	0.056	264.595
S_G - Slow Global	3.868	3.275	15367.328
E_G - Edge Global	0.446	0.043	17928.710

Fully Preprocessed Data

Source	RMS	% of A-var	Relative to IID
A - All	9.763	100.000	1.000
D - Fast	6.896	49.893	1.003
S - Slow	6.888	49.774	1.001
E - Edge	0.562	0.331	0.583
A_G - All Global	0.866	0.787	568.427
D_G - Fast Global	0.610	0.391	568.063
S_G - Slow Global	0.604	0.383	556.624
E_G - Edge Global	0.110	0.012	1632.983

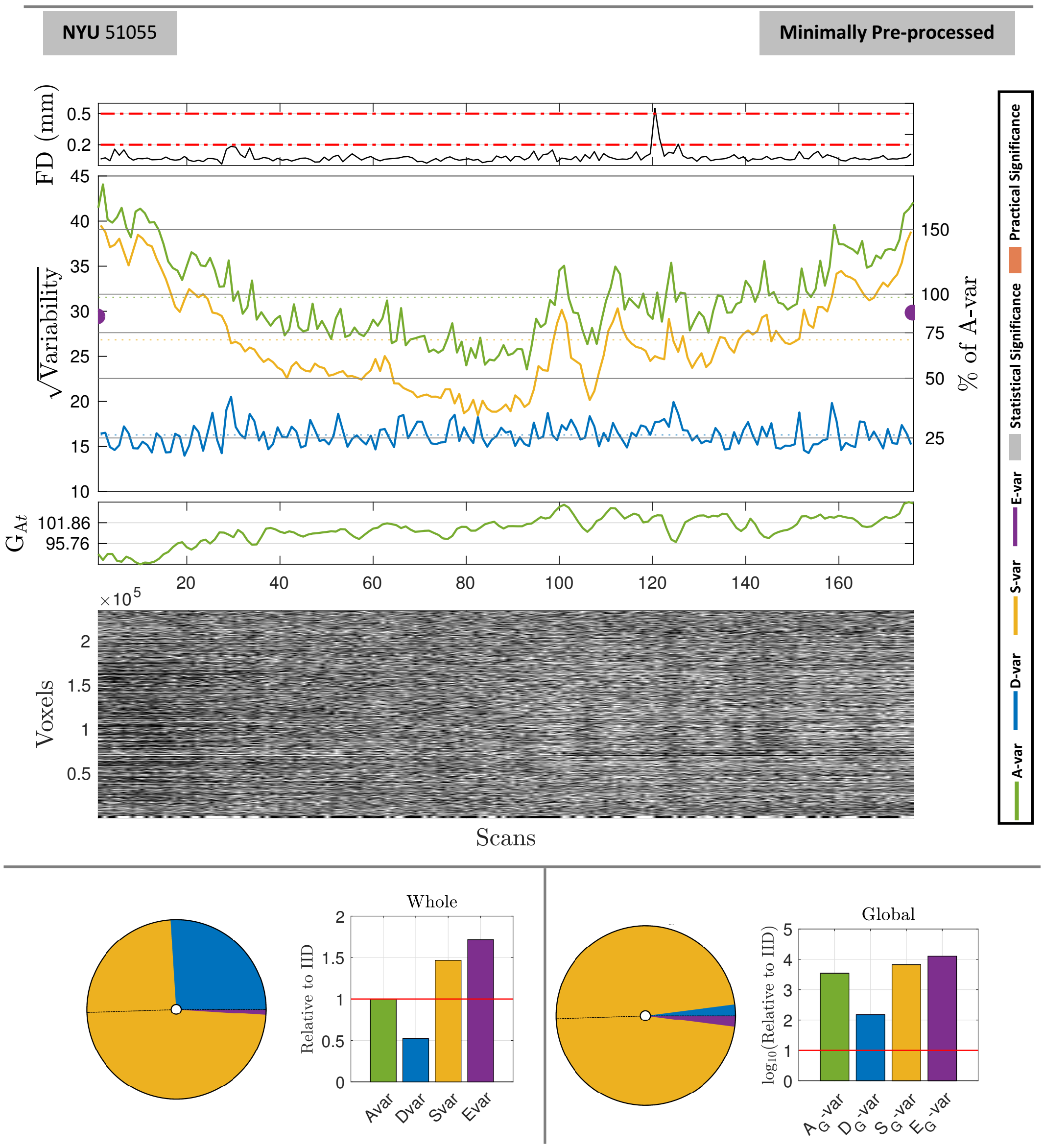

Figure S16: Illustration of DSE and DVARS inference for ABIDE-NYU 51055 minimally pre-processed. Layout as in Figure 5. Fast variability component only occupies 3/4 of the whole variability, while, fast and edge variance have very small share of the global variability.

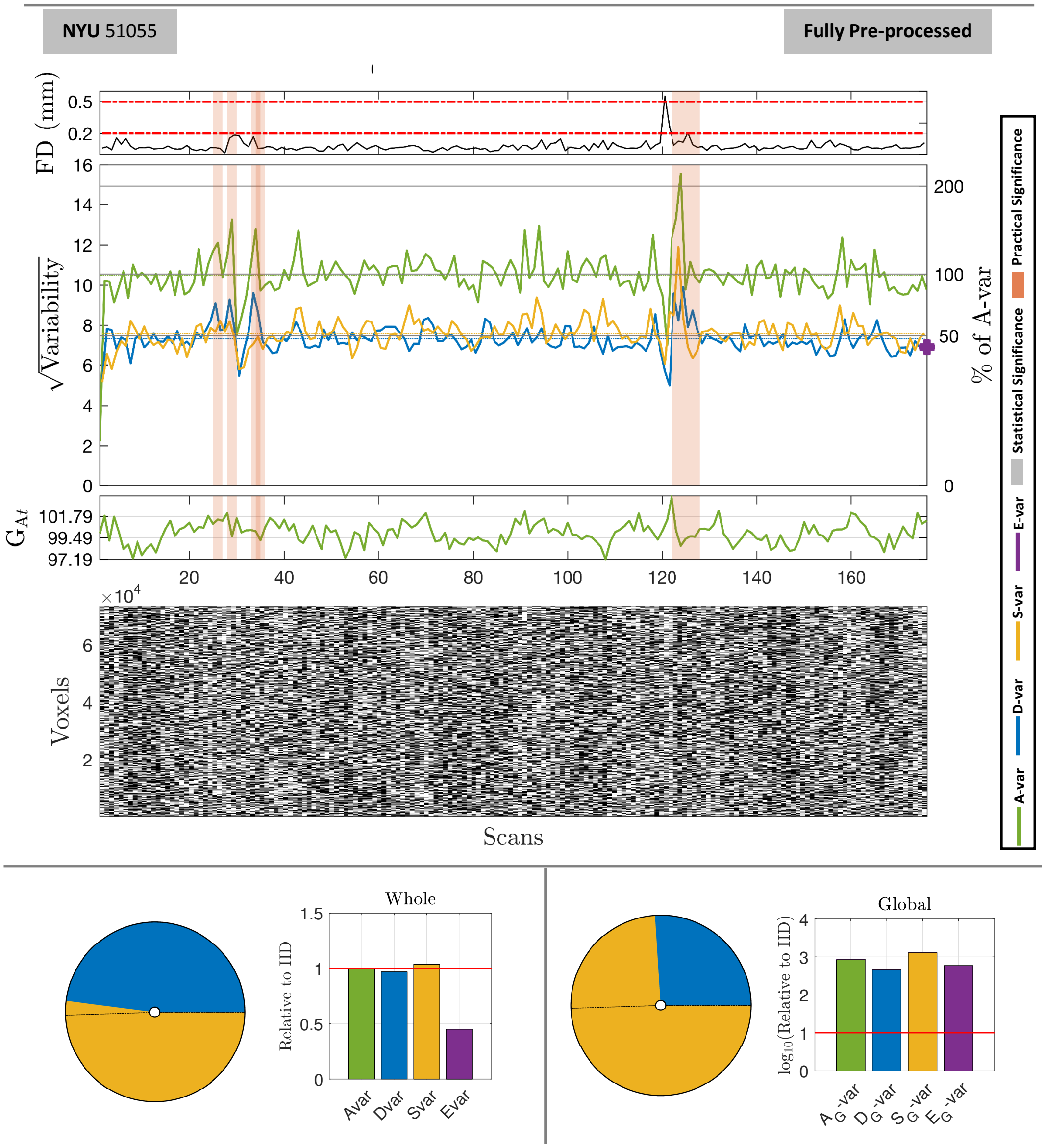

Figure S17: Illustration of DSE and DVARS inference for ABIDE-NYU 51055 fully pre-processed. Layout as in Figure 5. Given the descriptions for Figure S6, the significant spikes are mostly around the scans which the movement were regressed out in pre-processing due to incursion in FD. For example, compare the excursion in FD and S- and D-var around scan 30. Similar effect can also be seen around scan 120 to 125. The whole variability has almost fairly divided into two main components of fast and slow variabilities, whereas, the fast variability only occupies 1/4 of the global variability in this subject.

Table S7: DSE ANOVA Tables for ABIDE-NYU 51055. Minimally preprocessed data (top), fully preprocessed (bottom). The same pattern of changes, as subject 51050, can be seen in this subject in both whole and global variability components.

Minimally Preprocessed Data

Source	RMS	% of A-var	Relative to IID
A - All	31.893	100.000	1.000
D - Fast	16.278	26.050	0.523
S - Slow	27.244	72.974	1.467
E - Edge	3.149	0.975	1.716
A_{G} - All Global	3.884	1.483	3480.808
D_{G} - Fast Global	0.568	0.031	150.126
S_{G} - Slow Global	3.801	1.420	6705.578
E_{G} - Edge Global	0.560	0.030	12748.095

Fully Preprocessed Data

Source	RMS	% of A-var	Relative to IID
A - All	10.544	100.000	1.000
D - Fast	7.316	48.145	0.968
S - Slow	7.574	51.598	1.037
E - Edge	0.533	0.256	0.450
A_{G} - All Global	1.145	1.180	869.650
D_{G} - Fast Global	0.583	0.306	454.242
S_{G} - Slow Global	0.983	0.869	1288.208
E_{G} - Edge Global	0.071	0.004	594.008

7.2. Voxel-wise lag-1 Autocorrelation Coefficients for HCP

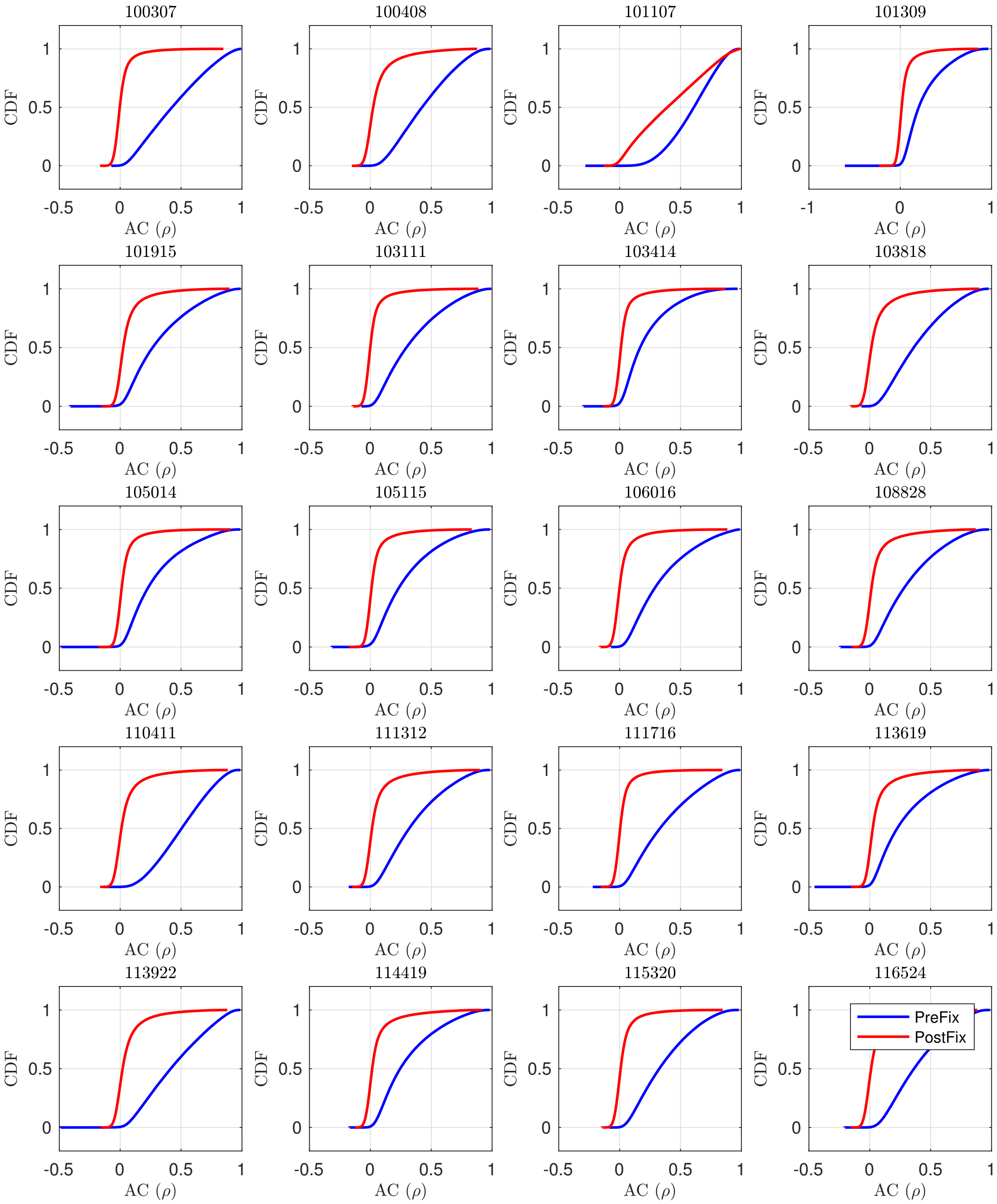

Figure S18: Cumulative Distribution Function (CDF) for lag-1 voxel-wise autocorrelation for the first 20 subjects of the HCP 100 unrelated package. Solid blue lines indicate lag-1 autocorrelation of subjects on MPP level (PreFix) while solid red lines indicate the lag-1 AC of subjects on the FPP level (PostFix).

7.3. DSE decomposition of Variability Images in Figure 11

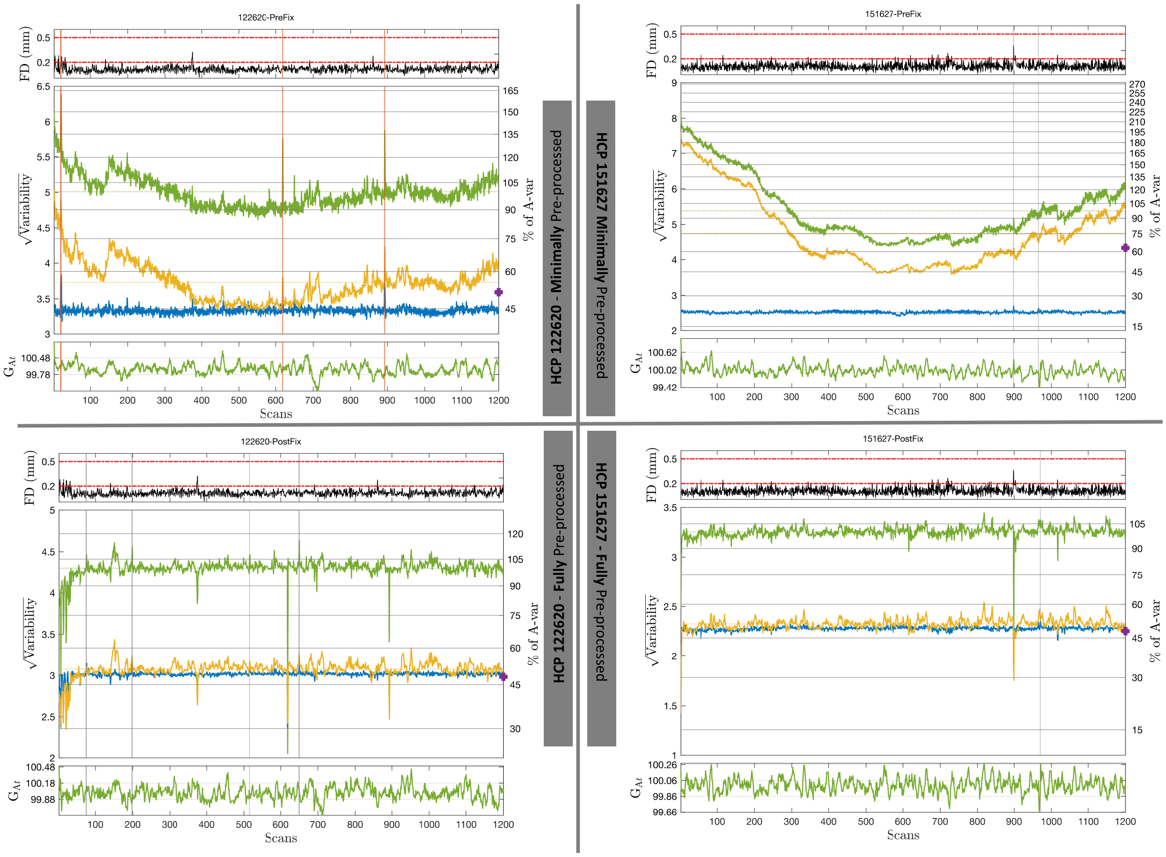

Figure S19: Each panel illustrates the FD (top sub-figure), the DSE decomposition (middle) and global signal (bottom) for two **"clean"** subjects found via DSE plots in figure 11 of the main text. For each subject, the results presented for MPP and FPP levels (top and bottom, respectively).

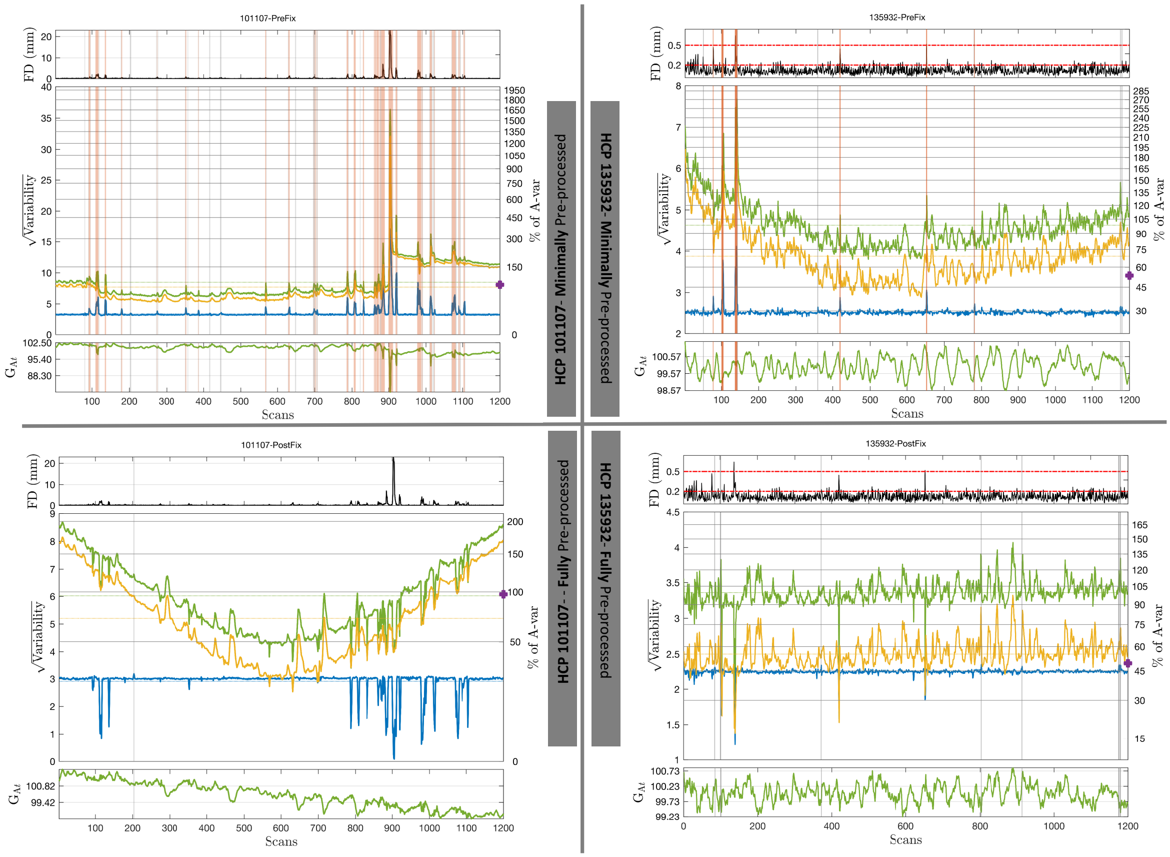

Figure S20: Each panel illustrates the FD (top sub-panel), the DSE decomposition (middle sub-panel) and global signal (bottom sub-panel) for two **"bad"** subjects found via DSE plots in figure 11 of the main text. For each subject, the results presented for MPP and FPP levels (top and bottom, respectively).

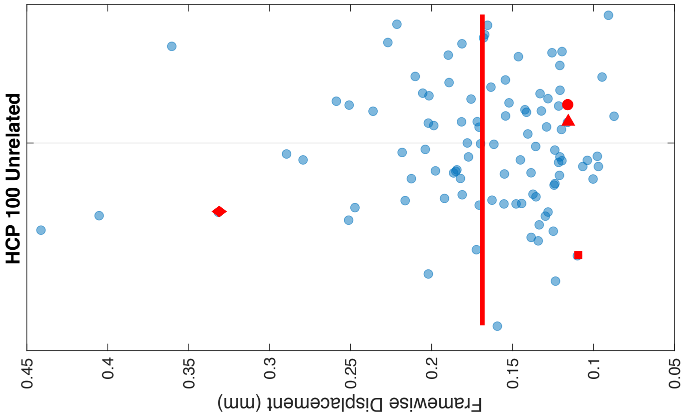

Figure S21: Framewise displacement of 100 subjects from the HCP cohort. Median is marked as vertical red line. FD of four subjects, 151627 (square), 122620 (arrow), 135932 (circle) and 101107 (diamond) has also flagged in red.

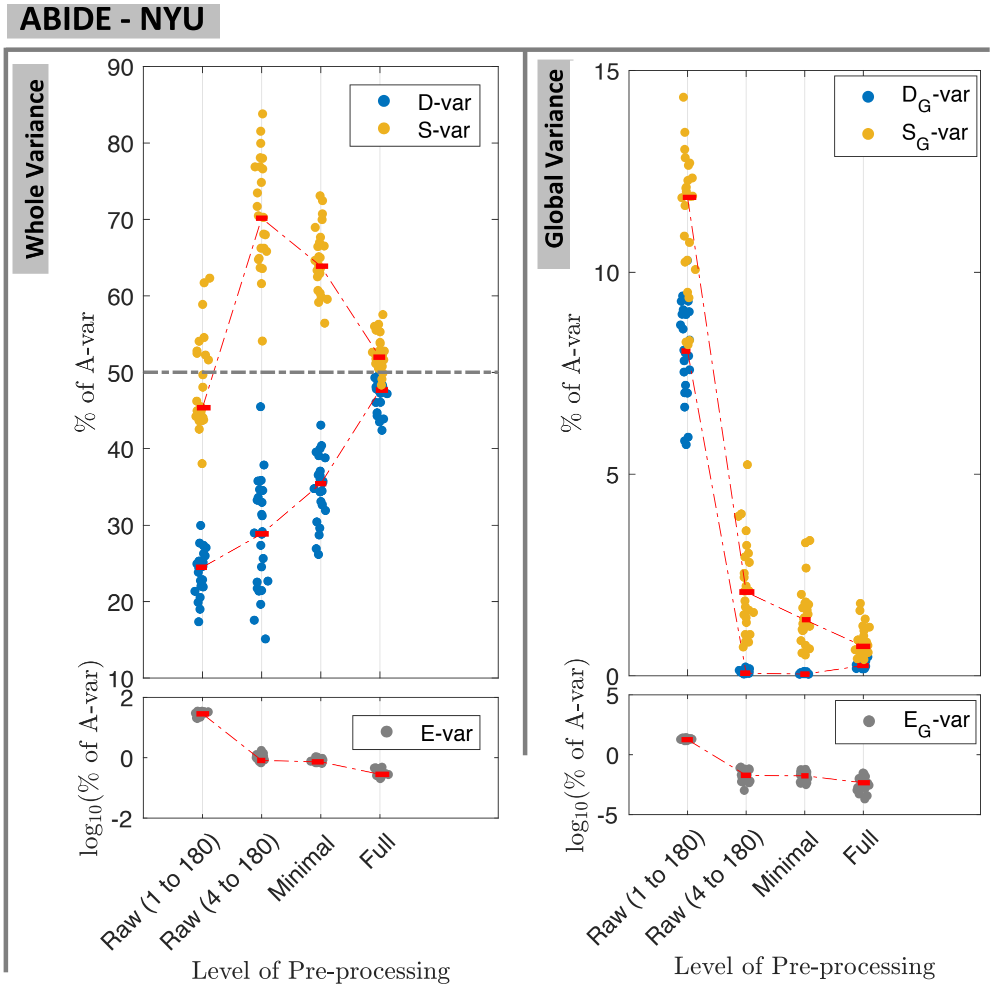

Figure S22: The DSE decomposition summary for ABIDE NYU. This follows a similar pattern to Figure 11 The results suggest how the DSE plots can be used to detect the possible magnetisation instability in early volumes of the ABIDE cohort and embolden the need of removing the first few volumes of subjects. A similar figure for 100 HCP subject is presented in Figure 11 of the main text.

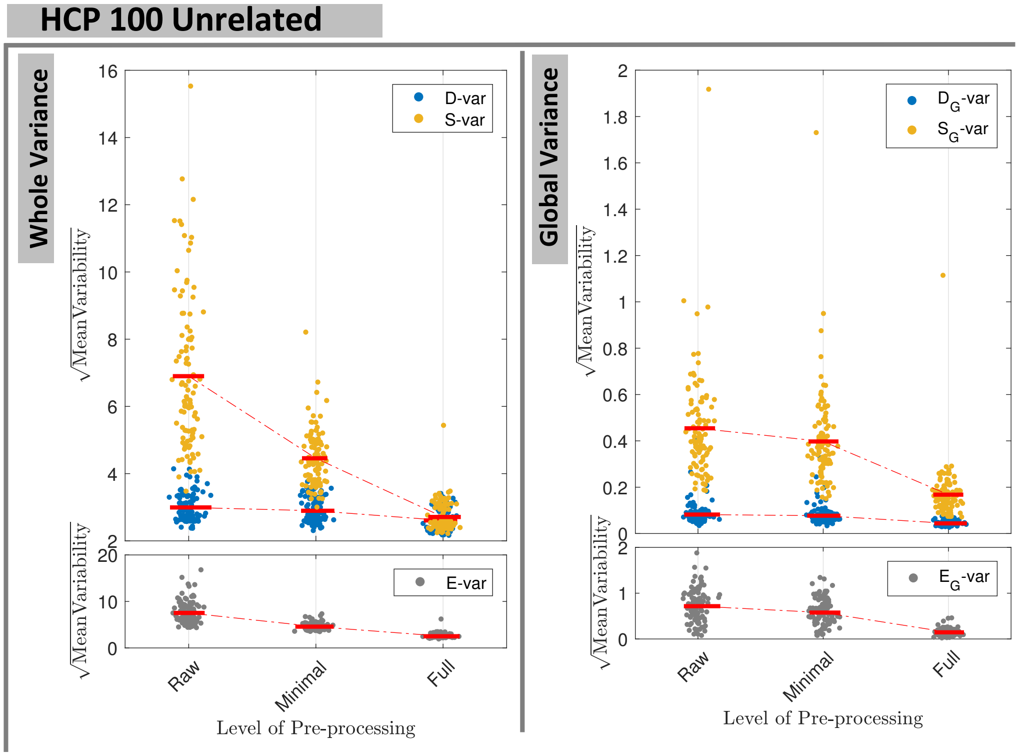

Figure S23: DSE decomposition summary for the HCP cohort without normalization by A-var. Reveals the relatively stable nature of D-var, and how successive preprocessing has greatest impact on S-var.

7.4. Variability Images and ICA components

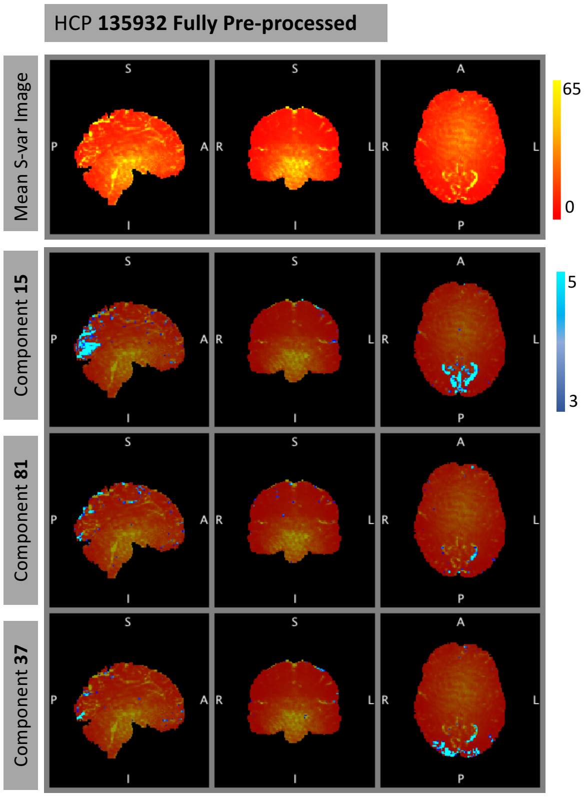

Figure S24: Three selected ICA components; 15, 81 and 37, classified as ”signal” or ”good” components via ICA-FIX overlied on the S-var image.

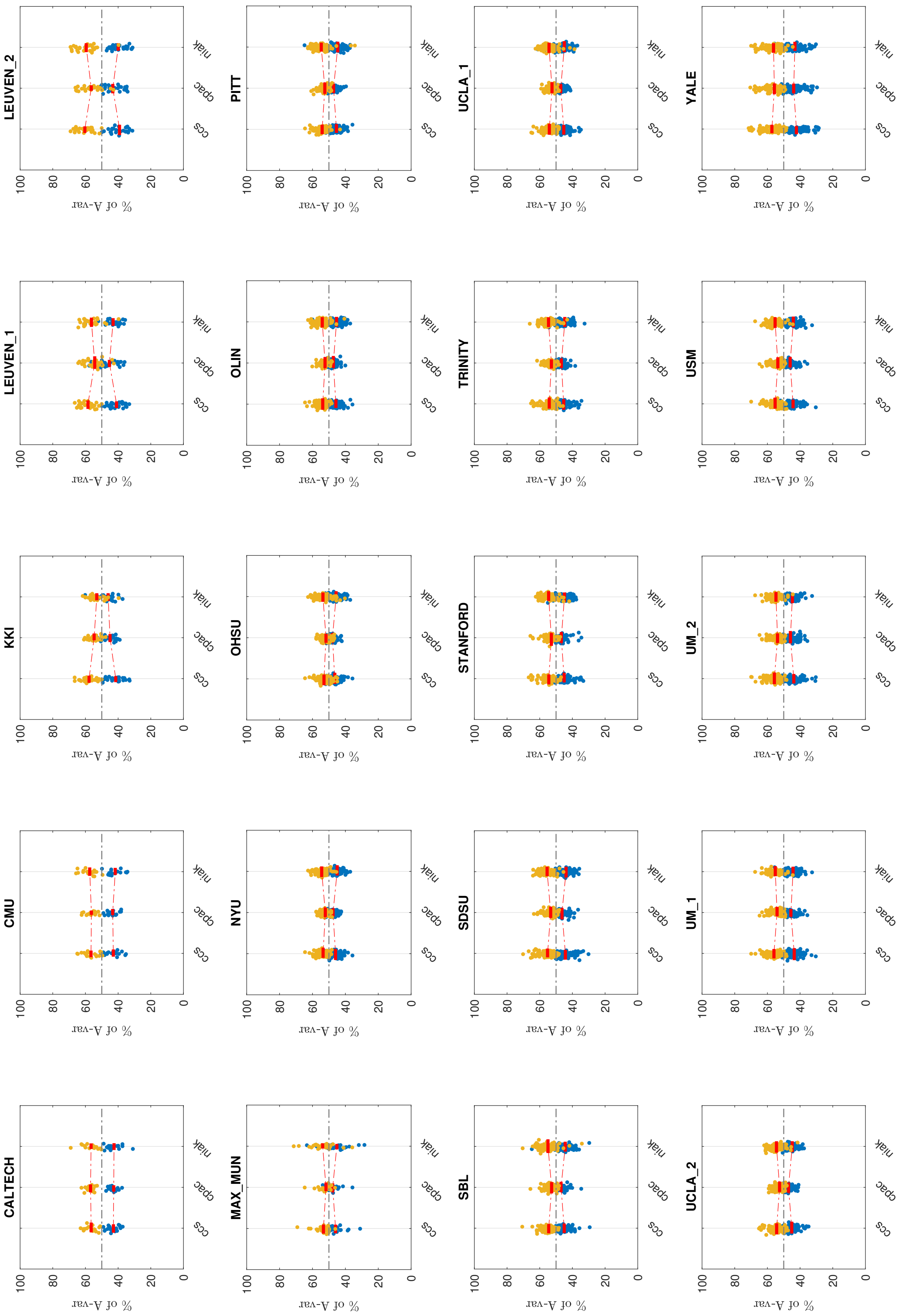

Figure S25: The DSE decomposition summary for 530 healthy subjects available across 20 acquisition sites in ABIDE dataset. The figure shows how the DSE decomposition can be utilised to compare the resting-state pipelines. The results suggest that the CPAC pipe-line might outperforms alternatives, CCS and NIAK.

References

Burgess, G.C., Kandala, S., Nolan, D., Laumann, T.O., Power, J.D., Adeyemo, B., Harms, M.P., Petersen, S.E., Barch, D.M., 2016. Evaluation of Denoising Strategies to Address Motion-Correlated Artifacts in Resting-State Functional Magnetic Resonance Imaging Data from the Human Connectome Project. Brain Connectivity 6, 669–680. URL: http://online.liebertpub.com/doi/10.1089/brain.2016.0435, doi:10.1089/brain.2016.0435.

Ciric, R., Wolf, D.H., Power, J.D., Roalf, D.R., Baum, G., Ruparel, K., Shinohara, R.T., Elliott, M.A., Eickhoff, S.B., Davatzikos, C., Gur, R.C., Gur, R.E., Bassett, D.S., Satterthwaite, T.D., 2016. Benchmarking confound regression strategies for the control of motion artifact in studies of functional connectivity. ArXiv URL: http://dx.doi.org/10.1016/j.neuroimage.2017.03.020, doi:10.1016/j.neuroimage.2017.03.020, arXiv:1608.03616.

Glasser, M.F., Sotiropoulos, S.N., Wilson, J.A., Coalson, T.S., Fischl, B., Andersson, J.L., Xu, J., Jbabdi, S., Webster, M., Polimeni, J.R., Van Essen, D.C., Jenkinson, M., 2013. The minimal preprocessing pipelines for the Human Connectome Project. NeuroImage 80, 105–124. URL: http://dx.doi.org/10.1016/j.neuroimage.2013.04.127, doi:10.1016/j.neuroimage.2013.04.127, arXiv:NIHMS150003.

Gordon, E.M., Laumann, T.O., Adeyemo, B., Huckins, J.F., Kelley, W.M., Petersen, S.E., 2014. Generation and Evaluation of a Cortical Area Parcellation from Resting-State Correlations. Cerebral Cortex URL: http://www.cercor.oxfordjournals.org/cgi/doi/10.1093/cercor/bhu239, doi:10.1093/cercor/bhu239.

Power, J.D., Barnes, K.A., Snyder, A.Z., Schlaggar, B.L., Petersen, S.E., 2012. Spurious but systematic correlations in functional connectivity MRI networks arise from subject motion. NeuroImage 59, 2142–2154. URL: http://dx.doi.org/10.1016/j.neuroimage.2011.10.018, doi:10.1016/j.neuroimage.2011.10.018.

Power, J.D., Cohen, A.L., Nelson, S.M., Wig, G.S., Barnes, K.A., Church, J.A., Vogel, A.C., Laumann, T.O., Miezin, F.M., Schlaggar, B.L., Petersen, S.E., 2011. Functional network organization of the human brain. Neuron 72, 665–78. URL: http://linkinghub.elsevier.com/retrieve/pii/S0896627311007926http://www.ncbi.nlm.nih.gov/pubmed/22099467http://www.pubmedcentral.nih.gov/articlerender.fcgi?artid=PMC3222858, doi:10.1016/j.neuron.2011.09.006.

Power, J.D., Mitra, A., Laumann, T.O., Snyder, A.Z., Schlaggar, B.L., Petersen, S.E., 2014. Methods to detect, characterize, and remove motion artifact in resting state fMRI. NeuroImage 84, 320–341. URL: http://dx.doi.org/10.1016/j.neuroimage.2013.08.048, doi:10.1016/j.neuroimage.2013.08.048, arXiv:NIHMS150003.
